# Supplementary material for: Detection and Verification of a Key Intermediate in an Enantioselective Peptide Catalyzed Acylation Reaction
Source: Molecules. 2022 Sep 26;27(19):6351. doi: 10.3390/molecules27196351 (PMC9571696; doi:10.3390/molecules27196351)
Supplement: Supplementary file 1 [file molecules-27-06351-s001.zip › molecules-1897819-supplementary.pdf]

Supplementary Materials

Detection and Verification of a Key Intermediate in an Enantioselective Peptide Catalyzed Acylation Reaction

Matthias Brauser, Tim Heymann and Christina Marie Thiele *

Clemens-Schöpf-Institute for Organic Chemistry and Biochemistry, Department of Chemistry, Technical University of Darmstadt, Alarich-Weiss-Straße 4, 64287 Darmstadt, Germany
* Correspondence: cthiele@thielelab.de

S1.0 Chemicals used 2

S2.0 Mass spectrometry 2

S2.1 ESI-MS measurements 2

S2.2 ESI-MS spectra 2

S3.0 NMR spectrometry 5

S3.1 Sample composition 5

S3.2 Instrumental equipment 5

S3.3 NMR sample preparation 5

S3.4 Experimental parameters 5

S3.5 NMR spectra 6

S3.6 Fit of NMR kinetics 10

References 12

S1.0 Chemicals used

The tetrapeptide Boc-L-(π-Me)-His-^{A}Gly-L-Cha-L-Phe-OMe **1** and (*R*,*R*)-*trans*-cyclohexane-1,2-diol (*R*,*R*)**-2** were obtained from the Schreiner group, Justus Liebig University Giessen and used as is. (*S*,*S*)-*trans*-1,2-cyclohexane-1,2-diol (*S*,*S*)**-2** , toluene-d_8 (in ampules, 99.8 atom % D), dichloromethane-d_2 (DCM-d_2) (in ampules, 99.5 atom % D), acetic anhydride **3** (99.5%), isobutyric anhydride **4** (97%) as well as trimethylacetic anhydride **5** (99%) were obtained from Sigma Aldrich, Germany and used as is.

S2.0 Mass spectrometry

S2.1 ElectroSpray Ionization Mass Spectrometry (ESI-MS) measurements

MS and MS^n experiments were carried out using an LTQ Orbitrap XL (Thermo Scientific, Bremen, Germany), which was controlled by Xcalibur version 2.1. 5 µL of the sample containing 1 mg/ml analyte was loaded into a glass needle that was pulled to a tip of ca. 1 µm orifice diameter with a P97 Flaming/Brown type micropipette puller (Sutter Instrument Co., Novato, CA, USA), starting from 1.2 mm thin-walled glass capillaries (World Precision Instruments, Friedberg, Germany). Ionization was performed using a home-built nano-electrospray source [1] that was coupled to the LTQ Orbitrap XL instrument. The spray voltage was set between 0.9 and 1.2 kV to obtain a stable spray with a flowrate of approximately 300 nL / min. For MS/MS analysis, the collision energy was increased until no respective precursor ion remained. Spectra were collected by averaging 30 scans in the mass range of 100-2000 m/z.

S2.2 ESI-MS spectra

Hereinafter experimental mass spectra mentioned in the main text are shown. In addition to High Resolution Mass Spectrometry (HRMS) spectra of tetrapeptide **1** (Figure S1), different acylated tetrapeptides in the positive ionization mode (Figures S2-S3), spectra of the mixtures of the acetylated *trans*-cyclohexane-1,2-diols with the tetrapeptide in the negative ionization mode (Figures S4, S5) are shown. Fragmentation spectra using the Collision-Induced Dissociation (CID) and Higher-energy Collisional Dissociation (HCD) technique on ions selected allow the identification of the tetrapeptide as part of the acylated tetrapeptides, the complex of the acetlyated *trans*-cyclohexane-1,2-diols with the tetrapeptide and observed adducts containing the tetrapeptide itself.

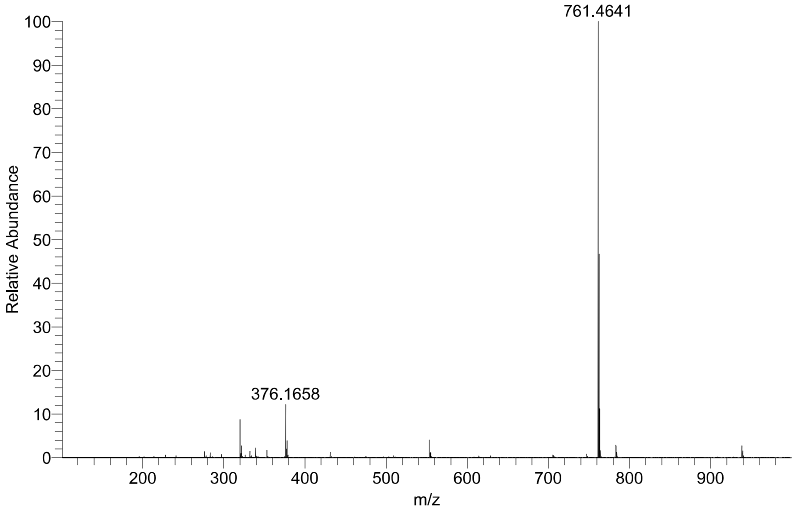

Figure S1. Positive ion mode (ESI^+) spectrum of tetrapeptide **1** sprayed from DCM. Mass of tetrapeptide **1** + H^+ with m/z = 761.4641 (theo: m/z = 761.4602).

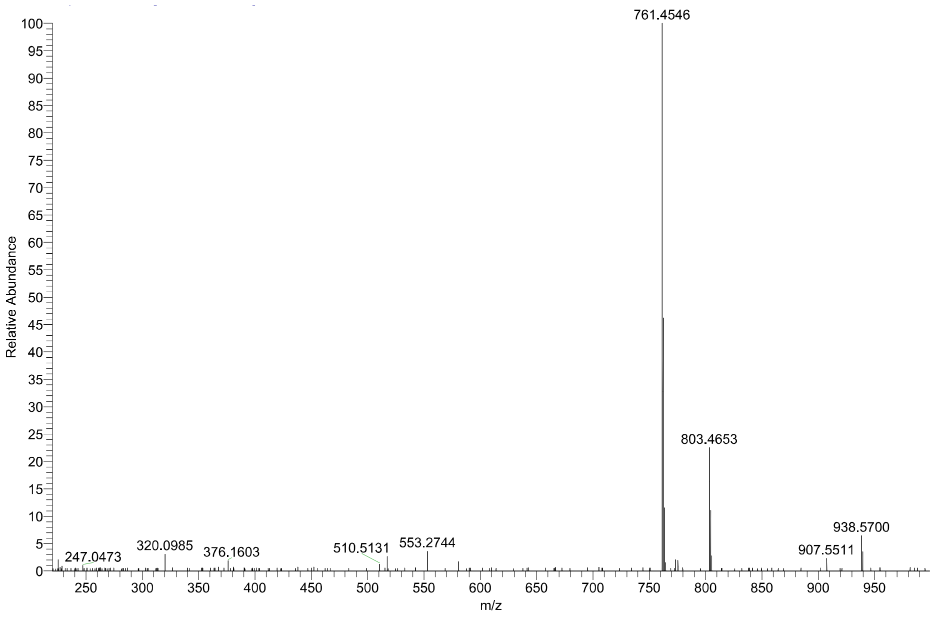

Figure S2. CID of m/z = 803.50 (**6**). Positive ion mode (ESI$^+$) spectrum of tetrapeptide **1** with acetic anhydride **3** sprayed from DCM. Mass of tetrapeptide **1** + H$^+$ with m/z = 761.4546 (theo: m/z = 761.4602) found as fragment.

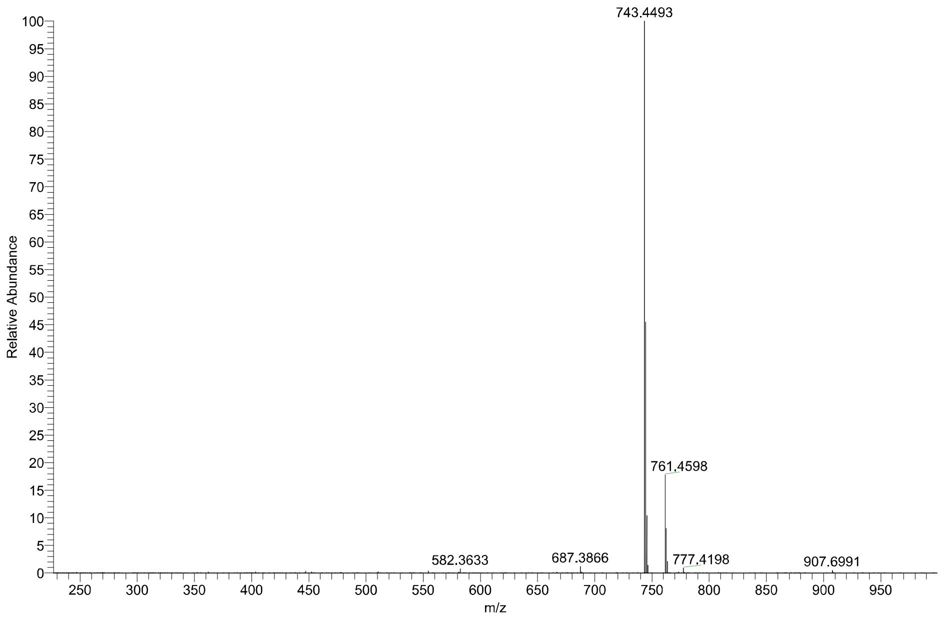

Figure S3. CID of m/z = 831.50 (**7**). Positive ion mode (ESI$^+$) spectrum of tetrapeptide **1** with isobutyric anhydride **3** sprayed from DCM. Mass of tetrapeptide **1** + H$^+$ with m/z = 761.4598 (theo: m/z = 761.4602) found as fragment.

To confirm the identity of the measured ions in the acylation experiments and proof that they are the result of acylation of tetrapeptide **1** using the different anhydrides, CID spectra in the positive ion mode were measured. If the resulting spectra contain the mass of tetrapeptide **1** + H$^+$ as a fragment, tetrapeptide **1** was part of the dissociated ion. The mass of tetrapeptide **1** + H$^+$ with theo: m/z = 761.4602 (Figure S1) was found as a fragment in the CID spectra of **6** (Figure S2) and **7** (Figure S3), thus confirming that they contain tetrapeptide **1.**

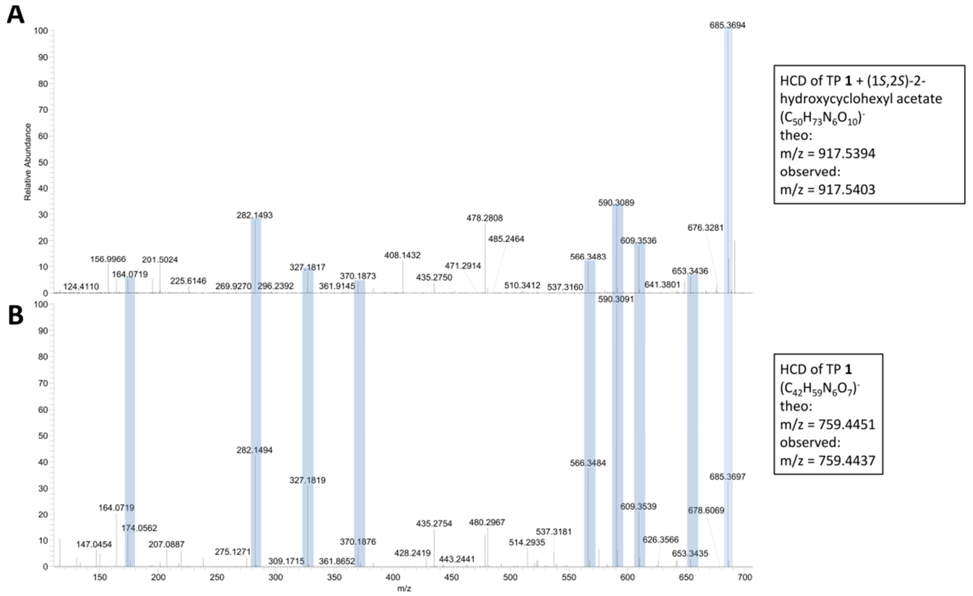

Figure S4. HCD of the reaction product complex with m/z = 917.53 (A) and HCD of **1** m/z = 759.44 (B) with equivalent fragment masses accentuated by light blue color. Stacked plot of negative ion mode (ESI$^-$) spectra of tetrapeptide **1** with acetic anhydride **3** as well as *S*,*S*-*trans*-cyclohexane-1,2-diol (*S*,*S*)-**2** (A) and **1** alone (B), sprayed from DCM.

Fragmentation of the reaction product complex of tetrapeptide **1** and the acetylated *S*,*S*-*trans*-cyclohexane-1,2-diol sprayed from DCM by HCD in the negative ion mode (ESI$^-$) reveals the same characteristic fragments as fragmentation of the corresponding reaction product complex with the acetylated *R*,*R*-*trans*-cyclohexane-1,2-diol **12**. As a result this shows that the detected complex also contains tetrapeptide **1**. Comparable fragment spectra of tetrapeptide **1** were obtained by fragmentation of the acetate complex of tetrapeptide **1** sprayed from toluene (Figure S5).

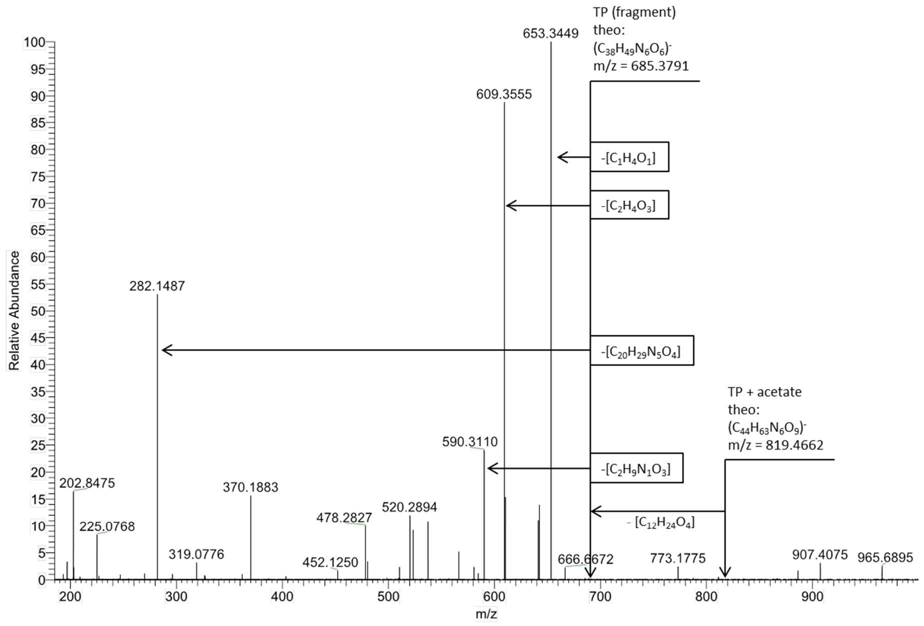

Figure S5. CID MS^3 of m/z = 819 (TP + acetate) and m/z = 685 (TP fragment). Negative ion mode (ESI$^-$) spectrum of tetrapeptide **1** with acetic anhydride **3**, acetic acid **15** as well as *R*,*R*-*trans*-cyclohexane-1,2-diol (*R*,*R*)-**2**, sprayed from toluene.

S3.0 Nuclear Magnetic Resonance spectrometry

S3.1 Sample composition

The composition of the samples used for the NMR measurements is shown in table S1. The excess of anhydride is calculated in relation to the tetrapeptide.

Table S1. NMR sample composition

sample	1	2	3	4	5	6
**mass** (Boc-L-(π-Me)-His-ᴬGly-L-Cha-L-Phe-OMe) / mg	9.85	11.64	4.85	4.90	5.50	5.40
**solvent**	DCM-d_2	DCM-d_2	DCM-d_2	DCM-d_2	DCM-d_2	Toluene-d_8
**mass** (solvent) / mg	510.40	749.00	652.80	596.00	686.90	384.70
**mass** (acetic anhydride) / mg	9.40	0	0	0	9.00	7.30
**mass** (isobutyric anhydride) / mg	0	3.71	0	0	0	0
**mass** (trimethylacetic anhydride) / mg	0	0	11.11	0	0	0
**mass** (acetic acid) / mg	0	0	0	2.00	0	0
**mass** ((1*R*,2*R*)-*trans*-1,2-Cyclohexandiol) / mg	0	0	0	0	1.00	0
**excess** (anhydride)	7.11	1.53	9.36	0	12.20	10.08

S3.2 Instrumental equipment

NMR experiments (^{1}H-^{13}C-Heteronuclear Single Quantum Coherence (HSQC) [2], ^{1}H-^{13}C-Heteronuclear Multiple Bond Correlation (HMBC) [3], Efficient Adiabatic SYmmetrized Rotating-Frame nuclear Overhauser Effect SpectroscopY (EASY-ROESY) [4] and 1D selective Nuclear Overhauser Effect (NOE) [5, 6]) were recorded on a Bruker Avance III HD 700 MHz spectrometer with a 5 mm QCIcryo probe (^{1}H/^{19}F, ^{13}C, ^{31}P, ^{15}N) equipped with a z-gradient coil with a maximum gradient strength of 53.0 Gcm^{-1} at corrected temperatures of 253 K (DCM-d_2) and 273 K (Toluene-d_8). For experimental parameters see S3.4.

S3.3 NMR sample preparation

The NMR samples were prepared by combining the chemicals listed under SI S3.1 in a 5 mm NMR tube. After addition of the solvent, the samples were thoroughly homogenized using a vortex mixer.

S3.4 Experimental parameters

1D selective NOE measurements

For the selective 1D-NOE measurements a pulse sequence with zero quantum suppression (selnogpzs) from Bruker's library was used [5, 6]. Selective refocusing for the signal of interest (O1P) was achieved by using RSnob pulses with their lengths set according to bandwidths of 60 or 70 Hz (calculated with the Bruker Shape Tool in TopSpin 3.2) depending on the dispersion of the signals [7]. The number of scans (NS) was set to 1024 and the relaxation delay (D1) was set to 2 s.

2D EASY-ROESY measurements

For the 2D EASY ROESY measurements the roesyadjsphpr pulse sequence from Bruker's library was used [4]. The spectra were acquired with a mixing-time of 200 ms, 64 scans, a relaxation delay (D1) of 1 s, a spinlock angle of 45° and an RF field of 6400 Hz.

S3.5 NMR spectra

In the following, experimental NMR spectra that are either additional spectra mentioned in the main text (Figures S6, S7, S8, and S15), full spectra of stacked plots (Figures S10-S14) or full 2D spectra used for cutouts in the main text (Figure S9), are shown.

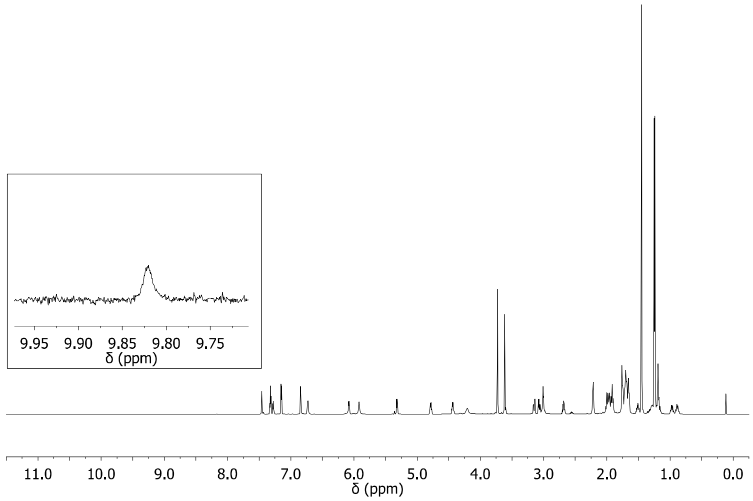

Figure S6. ^{1}H-NMR-spectrum of tetrapeptide **1** with isobutyric anhydride **4** in DCM-*d*$_2$ at -20 °C (700 MHz ^{1}H frequency). Insert showing a proton signal of an isobutylated-tetrapeptide **7** in the highlighted region.

Chemical shift changes and a new proton signal were detected in the ^{1}H-NMR spectrum of a solution of tetrapeptide **1** in DCM-*d*$_2$ by addition of acetic anhydride **3.** The same observation was made adding isobutyric anhydride **4** to a solution of tetrapeptide **1** in DCM-*d*$_2$ (Figure S6). No new proton signal in between 9 ppm and 10 ppm indicative of an acylated tetrapeptide was observed for the addition of trimethylacetic anhydride **5** to a solution of tetrapeptide **1** in DCM-*d*$_2$ (Figure S7). These findings are consistent with the low reactivity of **5** observed in reactions done by the Schreiner group [8].

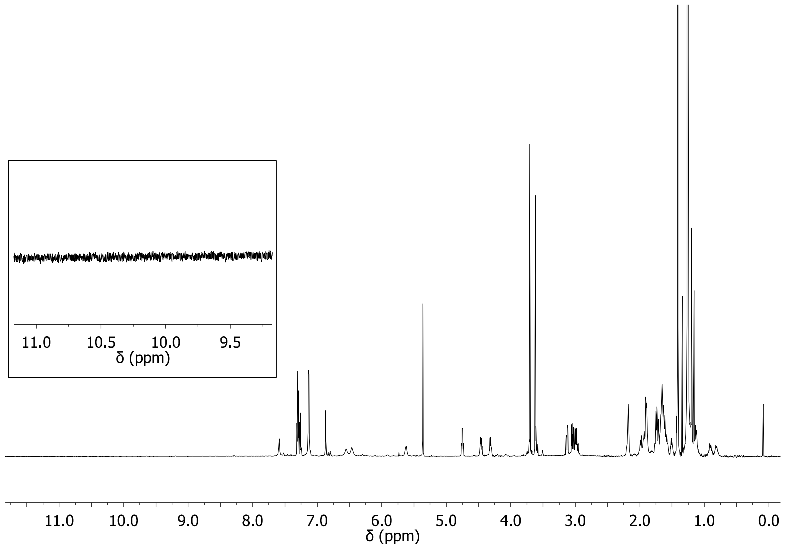

Figure S7. ^{1}H-NMR-spectrum of tetrapeptide **1** with trimethylacetic anhydride **5** in DCM-*d*$_2$ at -20 °C (700 MHz ^{1}H frequency). Insert showing no detectable proton signal of an acylated tetrapeptide in the highlighted region.

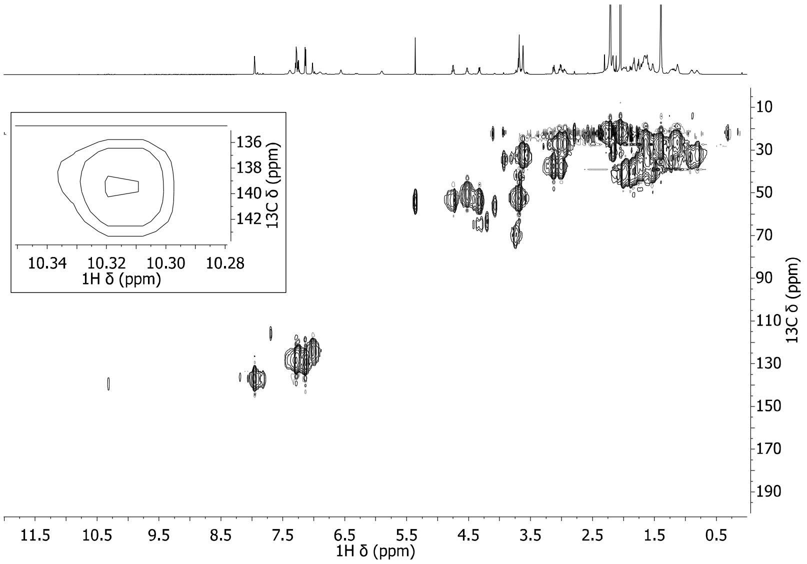

Figure S8. Full ^{1}H-^{13}C-HSQC-spectrum of tetrapeptide **1** with acetic anhydride **3** in DCM-*d*$_2$ at -20 °C (700 MHz ^{1}H frequency, 176 MHz ^{13}C frequency). Insert showing the correlation of H29$_{ac}$ and its respective carbon C29$_{ac}$.

To investigate the connectivity of the acetylated moiety ^{1}H-^{13}C-HSQC spectra were recorded for a solution of tetrapeptide **1** in DCM-*d*$_2$ with added acetic anhydride **3**. A correlation between a carbon chemical shift of 139 ppm and the proton signal of the acetylated π-methylhistidine moiety (H29$_{ac}$) can be seen (Figure S8) while carbon C29 of the unacetylated tetrapeptide **1** has a ^{13}C-chemical shift of 137 ppm in the same spectrum. This information can be used to correlate the carbon chemical shift of C29$_{ac}$ of the acetylated tetrapeptide **6** at 139 ppm and the chemical shift of the carbonyl carbon of the acetyl moiety over the methyl resonance Methyl$_{ac}$ using the ^{1}H-^{13}C-HMBC (Figures 6, Full spectrum S9). These correlations indicate a covalent bond between the π-methylhistidine moiety and the acetyl fragment.

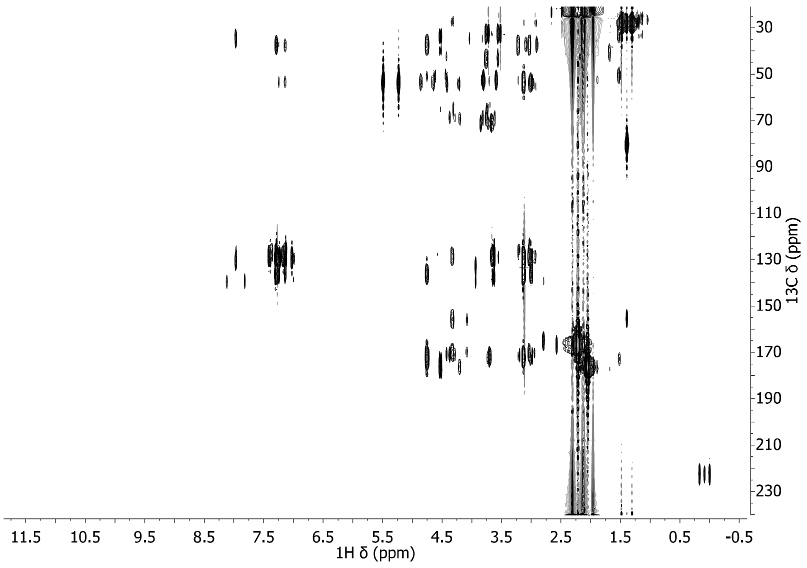

Figure S9. Full ^{1}H-^{13}C-HMBC-spectrum of tetrapeptide **1** with acetic anhydride **3** in DCM-d$_2$ at -20 °C (700 MHz ^{1}H frequency, 176 MHz ^{13}C frequency).

Further evidence for the connectivity of the acetyl fragment and the acetylated π-methylhistidine moiety of tetrapeptide **6** is found in recorded selective 1D-^{1}H-NOE spectra. The selective excitation at the resonance of H29 of the unacetylated π-methylhistidine moiety of tetrapeptide **1** (Figure S10) shows a correlation with $H29_{ac}$ at 10.5 ppm. The same correlation is found for the excitation of $H29_{ac}$ at 10.5 ppm (Figure S11).

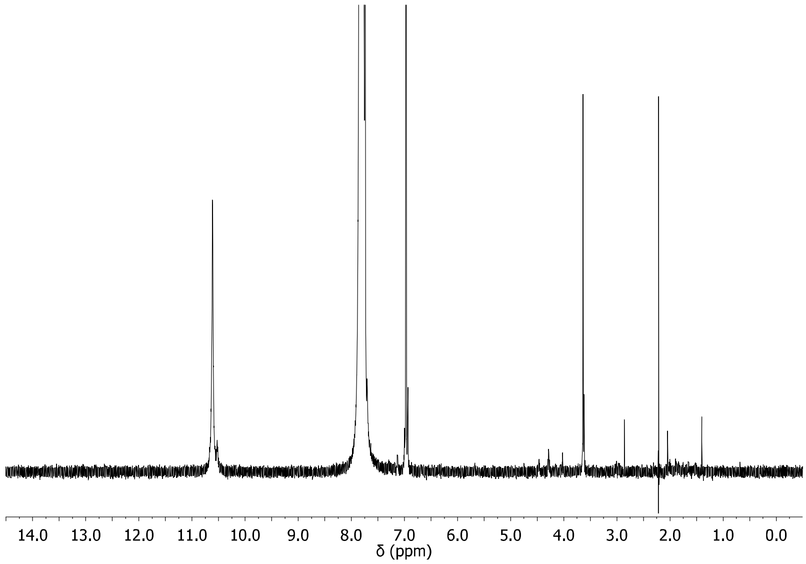

Figure S10. Selective 1D-^{1}H-NOE spectrum of tetrapeptide **1** with acetic anhydride **3** in DCM-d_2 at -20 °C (700 MHz ^{1}H frequency). Excitation at the H29 resonance at 7.8 ppm.

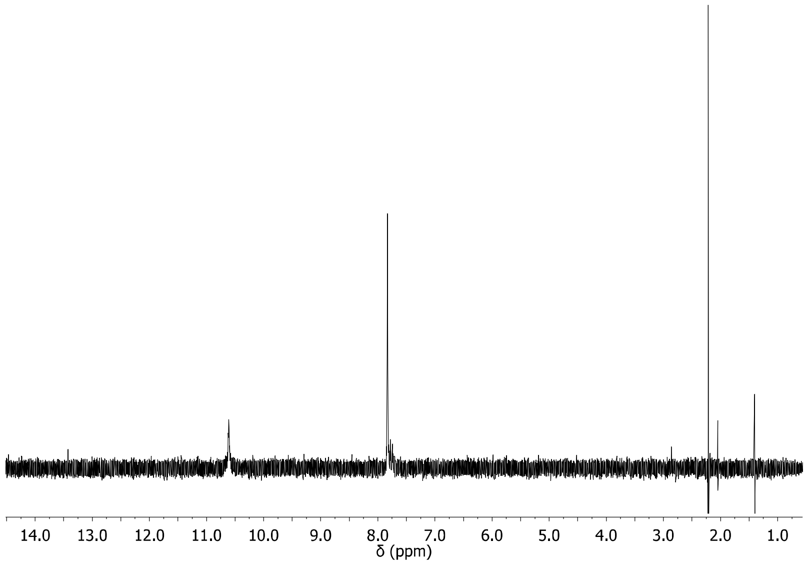

Figure S11. Selective 1D-^{1}H-NOE spectrum of tetrapeptide **1** with acetic anhydride **3** in DCM-d_2 at -20 °C (700 MHz ^{1}H frequency). Excitation at the $H29_{ac}$ resonance at 10.6 ppm.

For excitations at the $Methyl_{ac}$ resonance of the acyl fragment at 2.9 ppm correlations with $H29_{ac}$ of the acetylated tetrapeptide **6** and H29 of tetrapeptide **1** are found (Figure S12).

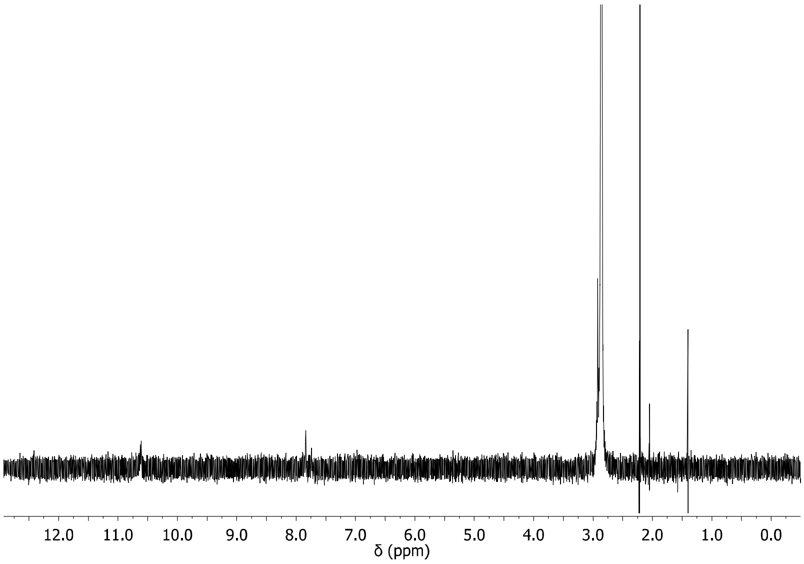

Figure S12. Selective 1D-^{1}H-NOE spectrum of tetrapeptide **1** with acetic anhydride **3** in DCM-d_2 at -20 °C (700 MHz ^{1}H frequency). Excitation at the Methyl$_{ac}$ resonance at 2.9 ppm.

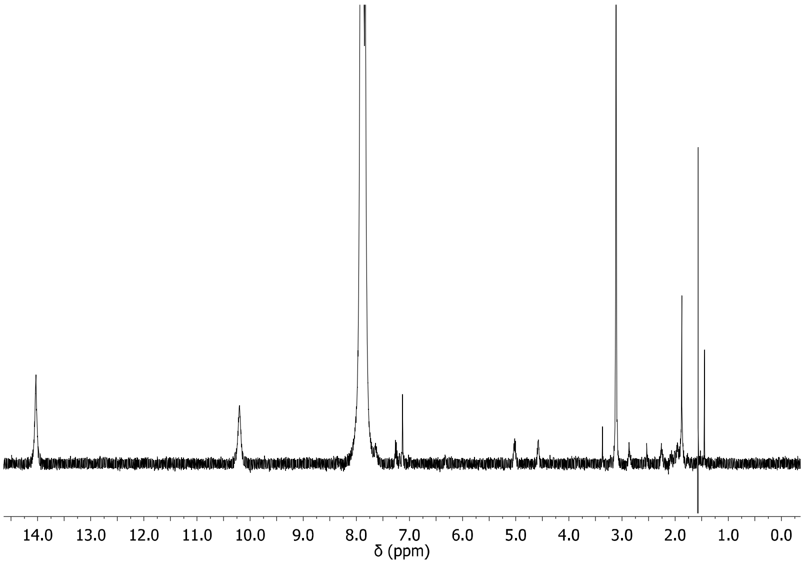

Figure S13. Selective 1D-^{1}H-NOE spectrum of tetrapeptide **1** with acetic anhydride **3** in toluene-d_8 at 0 °C (700 MHz ^{1}H frequency). Excitation at the H29 resonance at 7.9 ppm.

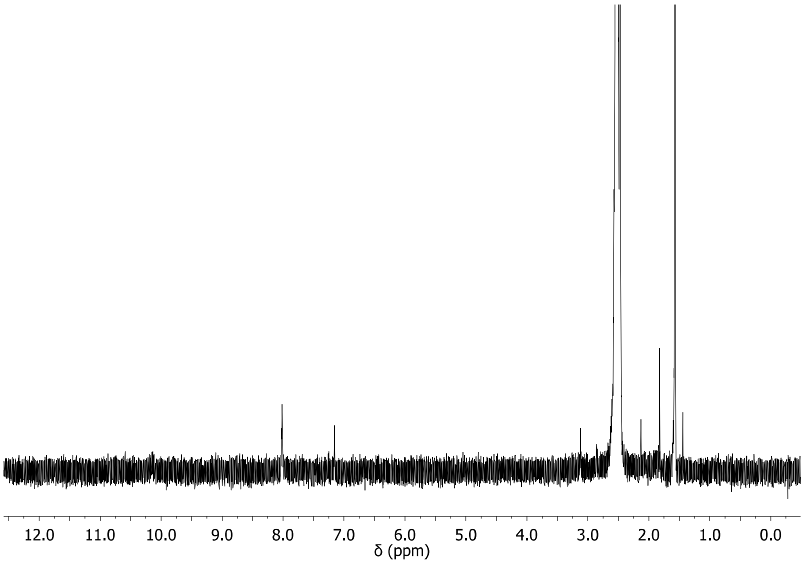

Figure S14. Selective 1D-^{1}H-NOE spectrum of tetrapeptide **1** with acetic anhydride **3** in toluene-d_8 at 0 °C (700 MHz ^{1}H frequency). Excitation at the Methyl$_{ac}$ resonance at 2.5 ppm.

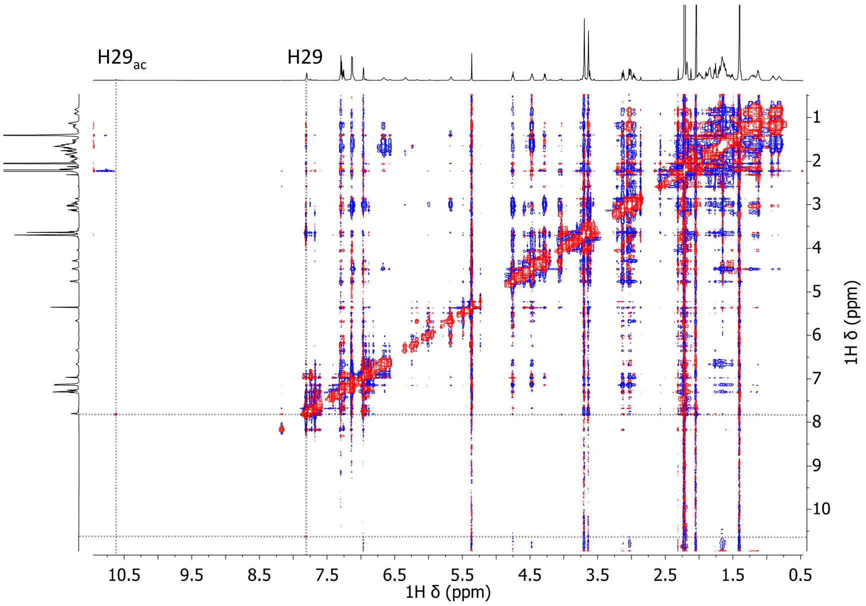

Figure S15. Full ^{1}H-^{1}H-EASY-ROESY-spectrum of tetrapeptide **1** with acetic anhydride **3** in DCM-d_2 at -20 °C (700 MHz ^{1}H frequency). Correlation of H29 and H29$_{ac}$ marked by lines. Spectrum was covariance processed [9].

The correlations found in DCM-*d*$_2$ solutions are also found in solutions of tetrapeptide **1** and acetic anhydride **3** in toluene-*d*$_8$ (Figures S13 and S14). NOE spectra alone cannot differentiate between correlations through space (NOE) or chemical exchange in the case of the tetrapeptide **1** at 700 MHz [10]. Thus, a ^{1}H-^{1}H-EASY-ROESY spectrum in which the cross relaxation does not change sign [11], due to its transversal nature, allows separation of these effects. The full spectrum is shown in Figure S15. Thus, it identifies the correlation of H29$_{ac}$ in the acetylated tetrapeptide **6** and H29 of tetrapeptide **1** as exchange between the two species in solution.

S3.6 Fit of NMR kinetics

To follow the acetylation of the diol using NMR, 5.5 mg tetrapeptide **1** and 9.0 mg acetic anhydride **3** (12.2 fold excess) were dissolved in 686.9 mg DCM-*d*$_2$ in an NMR-tube. A time series of 1D-^{1}H-spectra acquired as a pseudo 2D-spectrum was recorded after 1.0 mg *R,R-trans*-cyclohexane-1,2-diol (*R,R*)**-2** were added to the solution. The time series was integrated and the obtained integrals correlated with the concentration using the starting concentration of the compounds.

For the kinetic fit shown below we assumed the reaction to be first order using the following differential equations, which were solved numerically using DynaFit [12]:

$$\frac{\mathrm{d}}{\mathrm{dt}}[\mathrm{H1}] = -\mathrm{k}[\mathrm{H1}] \quad \text{(SEq 1)}$$

$$\frac{\mathrm{d}}{\mathrm{dt}}[\mathrm{H1}_{ac} + \mathrm{H1'}_{ac}] = \mathrm{k}[\mathrm{H1}] \quad \text{(SEq 2)}$$

Describing herein the change in concentration of proton H1 [H1] of the diol and the sum of the concentrations of protons $H1_{ac}$ and $H1'_{ac}$ [$H1_{ac}$+$H1'_{ac}$] of the acetylated diol with the reaction rate constant k.

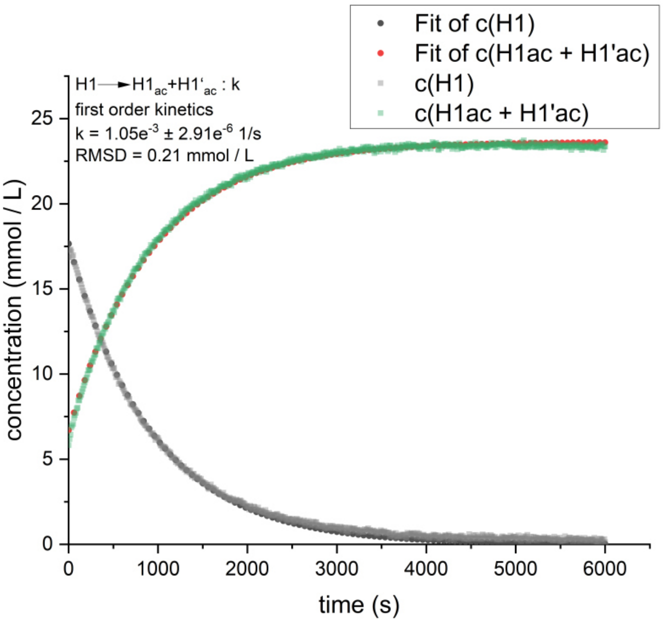

Figure S16. First-order kinetic fit using DynaFit [12].

For comparison a reaction of zeroth-order using SEq 3 (Figure S17) and a reaction of second-order using SEq 4 (Figure S18) were fit to the data using Origin (Pro), version number 9.8.0 [13] where $[\mathrm{H1}]_0$ is the starting concentration of H1 and c is an offset parameter. The lowest RMSD is still obtained for the first-order fit (RMSD = 0.21 mmol/L), while the zeroth-order fit with an RMSD of 2.44 mmol/L and the second-order fit with an RMSD of 0.46 mmol/L show larger deviations from the experimental data. This suggests a direct acetylation of the *R,R-trans*-cyclohexane-1,2-diol (*R,R*)**-2** by the acetylated tetrapeptide **6** under the conditions chosen.

$$[\mathrm{H1}] = [\mathrm{H1}]_0 - \mathrm{kt} \quad \text{(SEq 3)}$$

$$[\mathrm{H1}] = \frac{1}{\frac{1}{[\mathrm{H1}]_0} + \mathrm{kt}} + \mathrm{c} \quad \text{(SEq 4)}$$

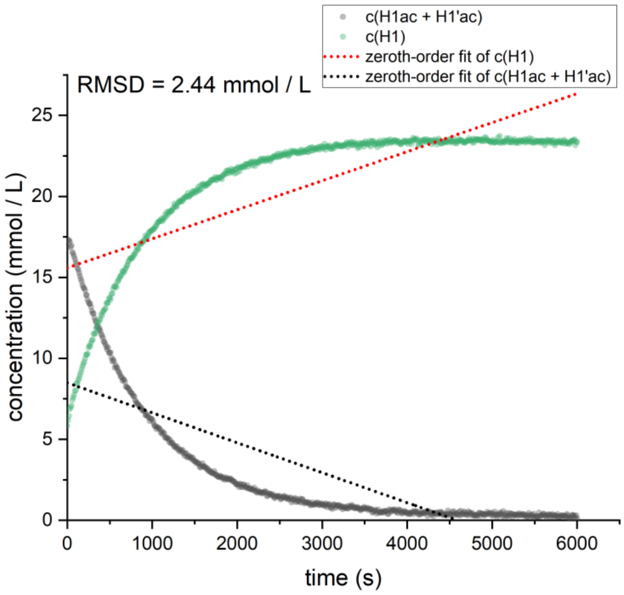

Figure S17. Zeroth-order kinetic fit (SEq3) using Origin (Pro), version number 9.8.0 [13].

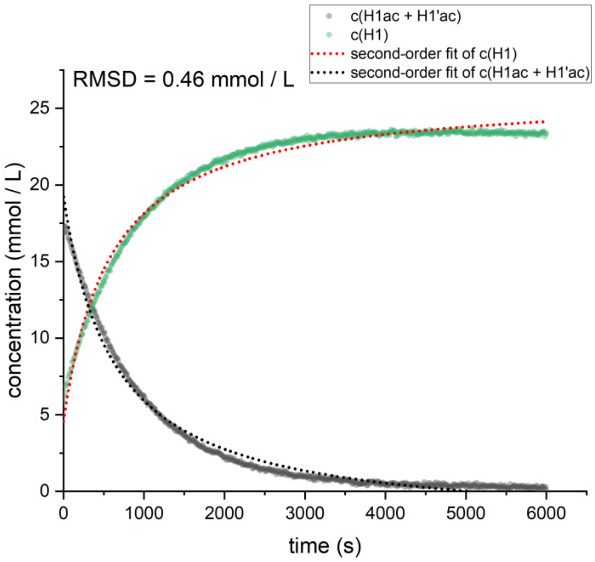

Figure S18. Second-order kinetic fit (SEq4) using Origin (Pro), version number 9.8.0 [13].

References

1. Nehls, T.; Heymann, T.; Meyners, C.; Hausch, F.; Lermyte, F., Fenton-Chemistry-Based Oxidative Modification of Proteins Reflects Their Conformation. *Int. J. Mol. Sci.* **2021,** 22, (18), 9927.
2. Bodenhausen, G.; Ruben, D. J., Natural abundance nitrogen-15 NMR by enhanced heteronuclear spectroscopy. *Chem. Phys. Lett.* **1980,** 69, (1), 185-189.

3. Bax, A.; Summers, M. F., Proton and carbon-13 assignments from sensitivity-enhanced detection of heteronuclear multiple-bond connectivity by 2D multiple quantum NMR. *J. Am. Chem. Soc.* **1986,** 108, (8), 2093-2094.
4. Thiele, C. M.; Petzold, K.; Schleucher, J., EASY ROESY: reliable cross-peak integration in adiabatic symmetrized ROESY. *Chem. Eur. J.* **2009,** 15, (3), 585-588.
5. Thrippleton, M. J.; Keeler, J., Elimination of Zero-Quantum Interference in Two-Dimensional NMR Spectra. *Angew. Chem. Int. Ed.* **2003,** 42, (33), 3938-3941.
6. Kessler, H.; Oschkinat, H.; Griesinger, C.; Bermel, W., Transformation of homonuclear two-dimensional NMR techniques into one-dimensional techniques using Gaussian pulses. *Journal of Magnetic Resonance (1969)* **1986,** 70, (1), 106-133.
7. Kupce, E.; Boyd, J.; Campbell, I. D., Short Selective Pulses for Biochemical Applications. *J. magn. reson., Ser. B.* **1995,** 106, (3), 300-303.
8. Müller, C. E.; Wanka, L.; Jewell, K.; Schreiner, P. R., Enantioselective Kinetic Resolution of trans-Cycloalkane-1,2-diols. *Angew. Chem. Int. Ed.* **2008,** 47, (33), 6180-6183.
9. Fredi, A.; Nolis, P.; Cobas, C.; Parella, T., Access to experimentally infeasible spectra by pure-shift NMR covariance. *J. Magn. Reson.* **2016,** 270, 161-168.
10. Procházková, E.; Kolmer, A.; Ilgen, J.; Schwab, M.; Kaltschnee, L.; Fredersdorf, M.; Schmidts, V.; Wende, R. C.; Schreiner, P. R.; Thiele, C. M., Uncovering Key Structural Features of an Enantioselective Peptide-Catalyzed Acylation Utilizing Advanced NMR Techniques. *Angew. Chem. Int. Ed.* **2016,** 55, (51), 15754-15759.
11. Bothner-By, A. A.; Stephens, R. L.; Lee, J.; Warren, C. D.; Jeanloz, R. W., Structure determination of a tetrasaccharide: transient nuclear Overhauser effects in the rotating frame. *J. Am. Chem. Soc.* **1984,** 106, (3), 811-813.
12. Kuzmič, P., Chapter 10 - DynaFit—A Software Package for Enzymology. In *Methods in Enzymology*, Johnson, M. L.; Brand, L., Eds. Academic Press: 2009; Vol. 467, pp 247-280.
13. OriginLab Corporation *OriginPro*, 9.8.0; OriginLab Northampton, MA, USA: 2021.
